# Stability Analysis through a Stability Factor Metric for IQRF Mesh Sensor Networks Utilizing Merged Data Collection

**DOI:** 10.3390/s24154977

**Published:** 2024-07-31

**Authors:** Gergely Sebestyen, Jozsef Kopjak

**Affiliations:** 1 Doctoral School of Applied Informatics and Applied Mathematics, Obuda University, 1034 Budapest, Hungary; 2 Kando Kalman Faculty of Electrical Engineering, Obuda University, 1034 Budapest, Hungary; kopjak.jozsef@kvk.uni-obuda.hu

**Keywords:** network stability, stability factor, merged data collecting, MDC, IQRF, FRC, TDMA, flooding routing, wireless sensor network

## Abstract

This paper introduces a novel stability metric specifically developed for IQRF wireless mesh sensor networks, emphasizing flooding routing and data collection methodologies, particularly IQRF’s Fast Response Command (FRC) technique. A key feature of this metric is its ability to ensure network resilience against disruptions by effectively utilizing redundant paths in the network. This makes the metric an indispensable tool for field engineers in both the design and deployment of wireless sensor networks. Our findings provide valuable insights, demonstrating the metric’s efficacy in achieving robust and reliable network operations, especially in data collection tasks. The inclusion of redundant paths as a factor in the stability metric significantly enhances its practicality and relevance. Furthermore, this research offers practical ideas for enhancing the design and management of wireless mesh sensor networks. The stability metric uniquely assesses the resilience of data collection activities within these networks, with a focus on the benefits of redundant paths, underscoring the significance of stability in network evaluation.

## 1. Introduction

Wireless sensor networks (WSNs) have rapidly expanded as a crucial technology in a variety of applications, including environmental monitoring, healthcare, and industrial automation, among others [1]. Strengthening reliable and stable communication in these networks, especially in areas where conditions can change and be challenging, is really important. In the face of changing environmental factors and possible interference, keeping the radio connections strong within the network is key to keeping communication smooth. When environmental issues challenge the stability of communication links between devices, not having backup options can lead to communication stopping, even if the devices are still working.

Pivoting toward the context of Low Power Wide Area Network (LPWAN) technologies, recent advancements have witnessed the emergence of options such as SigFox, NB-IoT, and LoRaWAN, each being designed and implemented with large coverage capabilities in the commercial market [2,3]. For instances wherein LPWAN communication cannot proffer optimal solutions, alternative technologies like the Wireless Local Area Network (WLAN) and Wireless Personal Area Network (WPAN), supplemented with an IoT gateway, have demonstrated their efficacy. Technologies including 6LoWPAN, Wi-Fi, Bluetoth Low Energy (BLE), and ZigBee, as well as WirelessHART and ISA100 in industrial contexts, have made notable strides in the market [4,5]. Furthermore, IQRF has emerged as a promising technology, providing effective solutions in the Internet of Things domain [6]. The recent literature has begun to explore the utility of IQRF in IoT applications, with several studies showcasing its potential and application [6,7,8,9,10,11]. Numerous applications in the field, primarily for monitoring purposes, have been built upon IQRF.

IQRF diverges from popular network solutions by not conforming to IEEE standards, yet it shows remarkable similarities with LR-WPAN networks that do, such as ZigBee systems based on the IEEE 802.15.4 standard [12]. Both IQRF and ZigBee feature mesh network designs, specific roles for network devices, and prioritize low energy consumption, despite their operational differences. IQRF uniquely employs Time Division Multiple Access (TDMA) for media access, allocating specific time slots to each device to enable organized and collision-free communication. This method enhances network stability and efficiency, especially in environments with numerous devices. Additionally, IQRF adopts a flooding routing protocol, where messages are broadcast to all neighboring nodes, ensuring high reliability in message delivery. This approach, while increasing network traffic, is effectively managed by the TDMA framework. In contrast, ZigBee, aligning with the IEEE 802.15.4 standard, uses Carrier-Sense Multiple Access with Collision Avoidance (CSMA/CA) for media access. This method involves devices sensing the channel before transmitting, aiming to minimize data collisions. For routing, ZigBee often relies on the Ad-hoc On-Demand Distance Vector (AODV) protocol, which establishes routes between nodes as needed, enhancing network traffic efficiency and adaptability [4,5,13,14,15].

Despite its potential as a wireless technology, both our experiences and existing research indicate that IQRF still presents areas needing more investigation, especially regarding network stability. Currently, there is no defined stability metric for IQRF networks. This paper aims to address this gap by introducing a novel stability metric specifically for IQRF networks, following the principles of how stability is defined for other networks. This new metric is designed to quantify the stability of IQRF networks and provide a standard for assessing and improving their performance.

IQRF employs directional flooding routing coupled with TDMA media access. In networks utilizing flooding routing, redundant paths may exist but these extra links between nodes do not always enhance the stability of network communication, particularly in terms of data collection. Based on experiences, there is a common belief in IQRF-based networks that increasing the number of network nodes will invariably improve redundant paths. However, superfluous devices might actually prolong communication times and reduce the battery life of the devices [16].

This paper aims to develop a specialized stability metric for IQRF networks, with a particular focus on data collection processes. Our proposed metric is designed to evaluate the resilience of data collection activities within these networks against temporary link disruptions. Emphasizing the criticality of stability in network assessment, this study particularly concentrates on networks engaged in Merged Data Collection (MDC) and Fast Respond Command (FRC) operations.

## 2. Related Works

IQRF technology, renowned for its robust documentation, is underpinned by a series of patents [13,17,18] that cover a diverse range of essential topics, crucial for the network’s functionality and operational efficiency. These patents provide a detailed exposition of the structural design of IQRF networks, offering insights into the intricate interconnections of devices. Such information is critical as it defines the network’s architecture, whether it adopts a mesh, star, or another layout, with each configuration presenting distinct advantages and challenges in terms of resilience and data transmission efficiency.

Focusing on routing mechanisms, the patents [13,19] explore advanced algorithms and methodologies essential for efficient data transmission across the network. These patents are instrumental in enhancing network performance, concentrating on optimizing data pathways to reduce latency, ensuring equitable distribution of network load, and strengthening overall communication reliability.

In the realm of data collection, patents [19,20] highlight innovative methods and technologies for collecting, processing, and transmitting data within the network. This segment is likely enriched with state-of-the-art advancements in sensor technology, data collecting and aggregation strategies, and energy-efficient solutions tailored for remote or battery-operated sensors. These patents are a testament to the depth of technical expertise and ongoing innovation characterizing IQRF network technology. For researchers and developers in this domain, an in-depth understanding of these patents is indispensable. It not only assists in circumventing potential legal infringements but also facilitates effective leveraging and expansion of the existing technological groundwork. In exploring the concept of network stability, the literature offers a diverse range of perspectives, particularly in the realm of wireless sensor networks.

This paper aims to synthesize these insights, focusing on the development of a tailored stability metric for IQRF networks. Our objective is to bridge the gap in current research by providing a clear and practical measure of stability specifically suited to the unique demands and characteristics of these networks. The concept of network stability in wireless mesh networks lacks a universally accepted definition. Typically, network stability is understood through various aspects such as node stability [21], link stability [22,23], path stability [21], routing stability [24,25], or topology stability [26], depending on the specific characteristics of the wireless network.

The current literature on IQRF networks does not sufficiently address network stability. However, detailed analyses of network and routing stability have been conducted for similar types of sensor networks. Such analyses could also be conducted for IQRF networks to enhance our understanding of their stability. Lim et al. [27] define stability in ZigBee wireless sensor network states that link stability and assert that it cannot be deterministically calculated due to the need to know the local positions of the communicating nodes, the geometry of the deployed environment, and the movements of mobile attenuators at all times. Given the impracticality of accounting for these factors constantly, link stability is instead defined as a varying probability. This approach involves considering variations in received signal strength between communicating nodes, which are accounted for as a Gaussian variable in the lognormal shadowing technique. This probabilistic definition recognizes the dynamic and unpredictable nature of link stability in real-world environments.

Sajadian et al. [28] defined stability as a weighted metric formula that incorporates three critical factors: the remaining energy of a node (E_n_), the received signal strength (RSSI), and the link quality (LQI). Each of these aspects is assigned a weight to reflect their varying importance. This variation depends on the network’s structure and the specific roles of the nodes. The remaining node’s energy indicates the operational lifespan and reliability, the received signal strength measures the quality of the connection in terms of signal power, and the link quality indicator evaluates factors like error rates and interference, affecting the stability and efficiency of the network link.

Lohs et al. [29] primarily focus on the challenges and characteristics of stability in wireless sensor networks. The study addresses the frequency and nature of unidirectional link occurrences and the dynamics of link changes within such networks. It emphasizes the prevalence and significance of unidirectional links, which are often more common than bidirectional links, and the need to consider these in routing protocols.

Delaney et al. [30] used link stability to evaluate and enhance the stability of tree-based routing structures. The stability is defined using two independent metrics: persistence and prevalence. Persistence refers to how long a route is likely to last before changing and prevalence relates to how often a particular route is chosen compared to other routes. A stable network is characterized as one that maintains routes that are both prevalent and persistent.

Ishibashi et al. [31] focus on evaluating and improving the stability of wireless sensor networks (WSNs) for data monitoring applications. It acknowledges the challenges posed by the inherent instability of wireless links in these networks and proposes a novel metric, the conditional data delivery ratio, for assessing network stability. The study also introduces an advanced local route re-selection mechanism aimed at enhancing the equitableness of routes to the central node, which is evaluated through simulations to demonstrate its effectiveness in improving network stability.

Xu et al. [25] introduced the “Link Stability Factor” (LSF) to assess the stability of a link in a mobile wireless sensor network. It is part of the Link Stability Based AODV (LSB-AODV) protocol. This metric is derived by estimating the distance between network nodes and comparing it to the maximum transmission range of these nodes. The basic idea is that shorter distances between nodes indicate more stable links. LSF is calculated by considering the strength of the received signal between nodes. This signal strength helps estimate how far apart the nodes are, using a model that predicts signal loss over distance. The more stable a link is perceived to be, the closer the nodes are thought to be. Furthermore, the overall stability of an entire network path is determined by examining the stability of each individual link within that path. The path’s stability is represented by the Path Stable Factor (PSF), which is influenced by the stability of its weakest link. The goal of this approach is to improve the reliability of network routing by favoring paths with more stable, and therefore likely more reliable, connections.

Based on a review of the literature in this field, stability in Wireless Sensor Networks (WSNs) emerges as a multifaceted and dynamic concept that could be assessed using various factors, including signal strength, battery life, connection quality, and data transmission efficiency. The prevailing view is that the environments in which WSNs operate are inherently unpredictable and constantly evolving, necessitating a flexible and comprehensive approach to defining and evaluating stability. In simpler terms, network stability refers to the consistency with which a network connection performs well without being overly susceptible to minor disturbances. This implies that a stable network or connection does not overreact to minor issues such as slight interference or small environmental changes. Essentially, a stable network maintains reliable and efficient performance without being overly sensitive to these minor fluctuations.

### Comparison of This Work

This paper introduces an analytical approach to assess the stability of IQRF network communication, focusing specifically on integrated data collection methods. We analyze the communication stability of IQRF networks using the graph network model described herein. To address observed communication stability challenges, we propose a novel metric for evaluating IQRF network communication stability. This metric quantitatively represents the robustness of communication, incorporating the redundancy of communication paths provided by the network’s flooding routing mechanism. A key aspect of our analysis involves exploring network redundancy metrics, which are essential for identifying the availability of alternative communication paths and data transfer routes among network devices. Such an evaluation is instrumental in assessing the stability of IQRF networks. Our approach includes a comprehensive examination, both quantitative and qualitative, of the network’s inherent redundancy. This research is crucial for assessing the network’s overall stability and robustness, especially when a flooding routing strategy is employed. By doing so, we aim to mitigate the risks of communication breakdowns and enhance the reliability and efficiency of these wireless sensor networks. Understanding the network’s stability also assists in identifying and addressing its vulnerabilities, particularly in terms of network interconnectivity.

## 3. Overview of Data Collection in IQMESH Networks

In the scope of Wireless Sensor Networks, integrated within the broader IoT framework, the utilization of IQRF technology represents a profound methodology for efficient and reliable data collection. This platform orchestrates an integration of transceivers, gateways, protocols, and auxiliary services, ensuring robust low-power wireless connectivity in sub-GHz Industrial, Scientific, and Medical (ISM) bands, spanning frequencies such as 433 MHz, 868 MHz, and 916 MHz. It strategically adopts a packet-oriented communication strategy, exhibiting a versatile application across varied wireless domains. The recent literature has presented numerous studies that explore the integration of IQRF technology within IoT applications [6,7,8,9,10,11]. IQRF, as a versatile technology, has been employed in a diverse array of fields. This spans a wide spectrum of applications, underscoring its utility and effectiveness in varied contexts. A particular focus resides on the IQRF DPA (Direct Peripheral Access) Framework and its innovative application of the Fast Response Command (FRC) [15,32]. The FRC optimizes a merged data collection method, meticulously exploiting the principles of non-optimal flooding routing to expedite data extraction from sensor nodes. Specifically, in a TDMA mesh network, the methodology initiates a request broadcast packet, which, upon receipt by the nodes, prompts each to transmit their respective data in designated time slots, akin to the dynamics of flooding routing. Each node seamlessly merges received packet payloads, embedding their measured value, culminating in the coordinator receiving a composite packet of synthesized sensor data, allocated in designated payload bytes.

Enhancements to this methodology are evident through IQRF’s data merging algorithm, incorporating an auxiliary non-routing packet transmission. Prior to network routing of the response, each node disseminates its data as a beacon message, allowing contiguous nodes to access the sensor data. This dualistic transmission, alongside regular routed packets, strengthens the reliability of data collection, particularly in fully discovered networks where response transmissions are duplicated. Critical to this method’s execution is its adherence to specified IQRF specifications, wherein larger packet sizes necessitate extended time slots, attributable to increased propagation times. Consequently, the methodology demonstrates a linear time complexity, O(N), with N denoting the network node quantity [1,16]. This confluence of robust wireless connectivity and meticulous data collection methodology underscores IQRF technology as pivotal within IoT frameworks, warranting further exploration and application across diverse wireless networking environments.

The IQMESH network utilizes flooding routing methodology. In flooding routing, instead of selecting the most optimal path, every route-selection network node replicates the message. Utilizing flooding route selection in the TDMA network can generate a robust and redundant route selection protocol but it may utilize more resources for message transmission than optimal solutions. Primarily suitable for fixed installation solutions due to its explicit TDMA medium access, network devices need to be paired with the network, throughout which each node is assigned a logical network address. Should these addresses be used for determining the packet repetition sequence during installation, meticulous placement based on logical addresses, which could be a problematic process, would be necessary. A simpler approach involves placing devices arbitrarily and determining the appropriate sequence of message repetitions based on the relative positions of the devices to each other, identified during a network mapping, and their neighboring proximity within their range.

**Figure 1:**
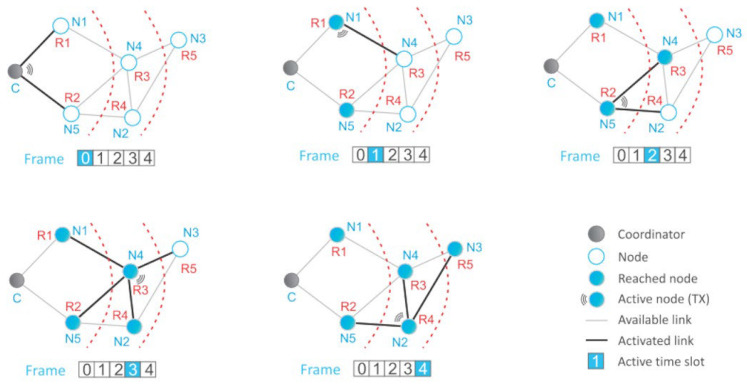
IQRF directional flooding process in each timeslot routing from C to node N3 [33].

In IQRF networks, nodes can be placed in a random order and logical addresses cannot be used to determine TDMA time slots for appropriate routing. The discovery process in IQRF networks ensures network routing, during which the network nodes are assigned a Virtual Routing Number (VRN). The VRN indicates the routing distance from the network coordinator and dictates dedicated time slots for nodes during routing. The coordinator is assigned a VRN of 0, while the network nodes are assigned VRNs starting from 1. Figure 1 shows a message delivery from C to N3 in the IQRF network. The flooding routing mechanism is illustrated with frames 0 to 4 representing the time slots assigned to nodes, with VRN numbers displayed in red text. According to the flooding routing mechanism, during each timeslot, the nodes that are active repeat the messages. Throughout the routing process, each node receives the message from the coordinator and forwards it during their designated time slot. After a successful transmission, all neighboring nodes will receive the message, indicated by it turning into a filled blue circle. Eventually, the designated node will receive the network message. Due to the properties of the flooding routing mechanism, every node will receive the message, even if it is not the addressed recipient.

The figure shows the VRN allocation by red color Rx and the logical addresses with blue Nx and three network zones. The zones within the network are also defined during discovery, with each zone’s number specifying the number of hops needed to reach a node within that specified zone. Every zone’s first node is the first node with the lowest logical address that identifies devices from the next zone. In IQMESH networks, routing addresses are assigned using a breadth-first search algorithm. In the TDMA network, each routing node repeats network messages to forward them to the addressee in their own time slot. If devices are not in proper order, network routing cannot function. The virtual routing sequence solves this problem. In the IQHMESH network, the DFM (Discovered Full Mesh) routing algorithm employs a virtual routing structure (VRS). During the discovery process, the coordinator assigns unique Virtual Routing Numbers (VRNs) to each routing device. In this approach, nodes initially receive logical network addresses and are assigned VRNs following the discovery process. The system organizes zones such that each contains nodes reachable from the coordinator within an equal number of hops. This structure enhances routing efficiency by determining the optimal order of nodes and enabling the construction of redundant links through the discovery process. Logical addresses serve users to identify the target node, while the IQMESH uses VRNs for routing to ensure appropriate time slots [15,34].

## 4. Network Communication Model in IQRF Networks

In the domain of wireless sensor networks, a prevalent challenge emerges from ensuring the stability of radio connections in the network. Specifically, in circumstances where network devices remain operational, environmental factors might instigate disruption in the wireless connection among two or more network entities, potentially crippling the communication infrastructure in the absence of redundant pathways. Within IQRF networks, devices can exploit multiple alternative routes, facilitated by a flooding-based route selection mechanism, to bolster the stability of network communication. In this context, network stability is intrinsically linked with the availability and reliability of these auxiliary and redundant paths. The flooding-based route selection algorithm enables the formulation of numerous alternative communication pathways among network devices, thereby enhancing network resilience. Through the strategic use of redundant connections, IQRF networks can mitigate the impact of communication link failures–attributed to environmental perturbations–and safeguard the continuity of network communication.

Our experiences with network deployment have highlighted the critical importance of incorporating multiple redundancies into the network. This is essential to guarantee continuous and unobstructed communication flow. The main issue we encountered was that the network’s multiple redundant paths often led to confusing stability patterns. To strictly evaluate the stability of IQRF networks, the crafting of relevant metrics and methodologies becomes necessary. One noteworthy metric is the ‘stability factor’, which will be employed in ensuing discussions to denote the minimum requisite number of redundant connections. Metrics gauging network redundancy are pivotal in inspecting the stability of IQRF networks. Such metrics encapsulate the quantification of redundant connections among network devices, factoring in the directionality of data transfer. Through the accurate measurement of network redundancy levels, the stability and resilience of the network—especially within the context of a flooding-based route selection mechanism—against communication disruptions can be carefully determined and optimized.

The topology of the wireless mesh network can be described by a directed graph in the form G(V, E), where

- Sensor devices are represented by the set of vertices, V(G);
- Communication links between sensor devices are established with the set of edges, E(G).

An edge e = (u, v) where u, v ∈ E exists between two devices u and v in the network if and only if both devices can receive each other’s transmitted packets. This means device u must be within the communication range of device v and device v must be within the communication range of device u. This requirement is due to the nature of IQRF networks. In these networks, all devices use the same IQRF hardware, are configured identically, and have consistent transmit power and receive sensitivity across all nodes. Because of these uniform characteristics, if device u can transmit packets that device v can receive, then device v can also transmit packets that device u can receive. Therefore, an edge e = (u, v) exists only if there is mutual communication capability between the devices, ensuring symmetric transmission and reception. The transmit power and receive sensitivity are consistent across all network nodes. The set of neighbors for a given node v can be represented as N1(v) = {u: (v, u) ∈ E}. The graph representing the network is labeled using graceful labeling; that is, the vertex labels range between 0 and |V| natural numbers. This labeling corresponds to the VRN addresses used in the wireless network. In the graph representing the network, vertices are denoted by u_i_ where i = VRN [33,35,36].

Due to this labeling, the wireless mesh network can be described in terms of directed graphs. Due to the use of the flooding route selection method, different graphs emerge for the transmission of request and response messages. The beacon phase ensures neighboring devices receive each other’s messages; therefore, for the complete merged data collection procedure, the graph can be considered undirected. If we abstract the network into a graph, the complete network map can be described using the graph’s adjacency matrix, representing the wireless sensor network [37].

The graph representation of the network topology can vary depending on the message transmission approach. Due to the direction of message flow, both the MDC and FRC processes involve multiple steps, leading to different graph representations. The merged data collection method begins with a broadcast message. Once the last node receives this message, it sends a response in its designated time slot and subsequent nodes continue to transmit data in their allotted time slots. In the IQRF FRC solution, nodes transmit their data to neighboring nodes through an additional non-routing packet transmission prior to sending it back. This transmission allows neighboring nodes to receive each other’s sensor data [14,16,37,38,39,40].

To describe the graph representation, we first needed to examine the following steps:

- Request via broadcast message;
- Beacon phase;
- Response message.

### 4.1. Request Broadcast Message

**Figure 2:**
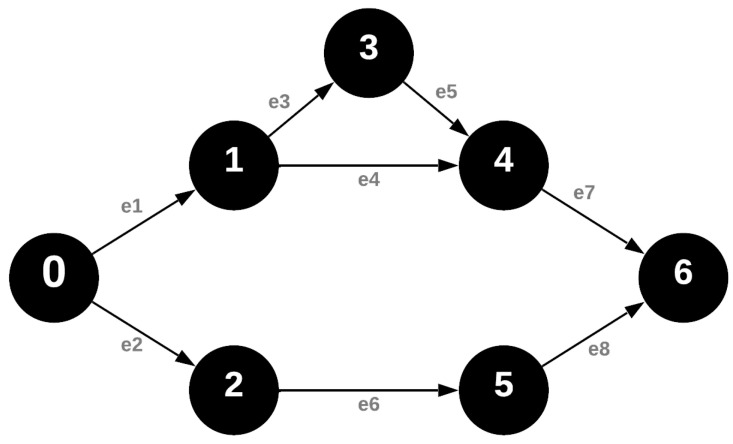
Graph representation of the network during the request broadcast phase of data collection.

The transfer of messages across network nodes in the FRC process commences with a request broadcast message. In TDMA networks, the flooding route selection process is based on the ascending order of the VRN numbers of the nodes discovered within the network. This ascending order is critically important for the proper operation of the directional flooding route selection mechanism. Consequently, in the graph representation of the network topology, every edge must originate from a vertex with a smaller VRN number and point to a vertex with a larger VRN number. The resulting graph is a directed acyclic weakly connected graph, which does not contain any directed cycles and is commonly referred to as a Directed Acyclic Graph (DAG) [36]. It is termed directed because there is a direction to the flow of messages and acyclic as the flow of messages can only proceed from nodes with smaller VRN numbers to those with larger VRN numbers. The allocation of increasing VRN numbers moving away from the coordinator is ensured by the breadth-first search during the initial network discovery process, as shown in Figure 2 [15,25,38].

In the IQRF network, a node can receive the request message for routing toward any node whose VRN number is smaller. The given node can receive after its own timeslot but afterward cannot route it. This is important to note because the message can be received after the nodes’ timeslot but only for processing it and not routing it.

### 4.2. Beacon Phase

During the beacon phase, nodes switch to receiving mode and each node transmits its beacon message within its designated time slot according to their VRN. It is crucial to note that this phase, implemented by the IQRF FRC, is not mandatory for the MDC method. From the perspective of the graph model representing the network, the beacon phase can potentially introduce network redundancy by dispatching an additional message in a network where every node functions as a router.

**Figure 3:**
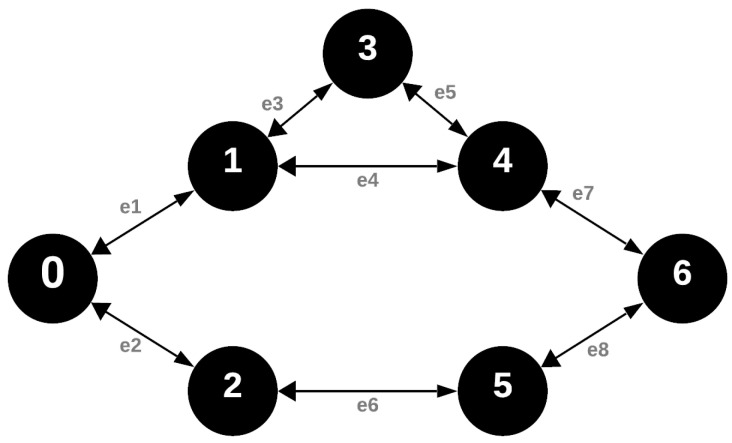
Graph representation of the network during the beacon phase of data collection.

Building on our assumptions, we understand that the sensors within the wireless sensor network exhibit homogeneity. This means every sensor shares identical characteristics and functionalities. For further emphasis, it is pivotal to note the symmetrical nature of the radio coverage among these sensors. In simpler terms, if one sensor can receive a message from another, the reverse is equally true. This foundational assumption ensures consistent and mutual communication capabilities throughout the network. As depicted in Figure 3, while the resulting graph is directed, the sharing of data among neighbors ensures that communication remains bidirectional.

### 4.3. Response Message

In the merged data-collecting process, the way the response message is put together is very similar to how the request message that was sent earlier is structured. Although they share this similar structure, there is a key difference in where these messages start. Usually, the response message begins at the node that is at the very edge of the network. This node can often be recognized because it has a noticeably higher VRN number. As this response message travels through the network, each node it meets is crucial. These nodes do more than just pass the message along. They actively combine the data they gathered earlier with the new information in the response message. After this combination, the improved message is then sent forward, following the specific time slot assigned to each node.

**Figure 4:**
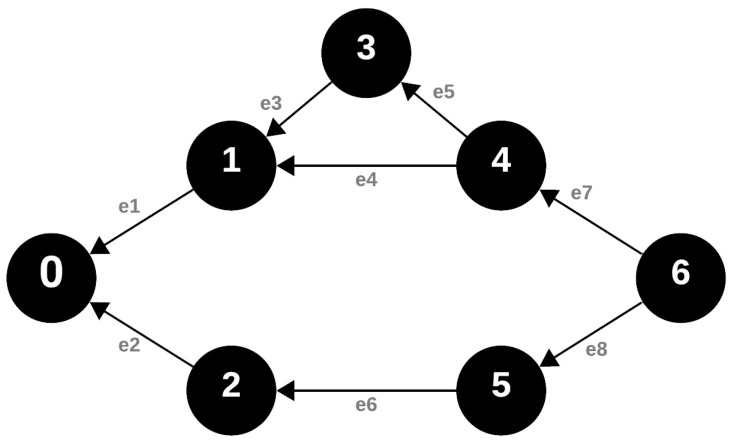
Graph representation of the network during the response phase of data collection.

Diving deeper into the mechanics of message transmission, it is clear that the response message has many similarities with the forwarded request message. Yet, there is an interesting detail when looking at how these messages work. The forwarded request message follows a sequence where the VRN (Versioned Routing Number) values increase step by step. In contrast, the response message reverses this pattern, following a sequence where the VRN values decrease or follow a different order. Figure 4 clearly shows that the response message follows a sequence with decreasing VRN numbers. This pattern is not just a minor detail in the process. It is important when looking at the highest level of incoming connections during the transmission of the response message. This level, or degree, acts as a measure. It shows how often a node receives messages from neighboring nodes that have higher VRN values.

## 5. Network Model of IQRF

Describing the IQRF mesh network model in a mathematical context involves formalizing its components and operations using mathematical concepts and notations. We focus on the centralized merged data collection from the network. The network can be modeled as a directed graph G = (V, E), where V represents the set of vertices (or nodes) in the network and E ⊆ V × V represents the set of directed edges (or links) between these nodes. Node v_0_ is the network coordinator and v_i_ ∈ V can be a router node or end device without routing capabilities. The existence of an edge (v_i_,v_j_) ∈ E signifies a direct communication pathway between nodes v_i_ and v_j_ [41].

### 5.1. Bonding Process

Let B:V→{0,1} be a bonding function, where B(v_i_) = 1 if node v_i_ is bonded to the network and B(v_i_) = 0 otherwise. The bonding process involves changing B(v_i_) from 0 to 1 using one of the bonding methods. After bonding, each node v_i_ is assigned a unique logical address L(v_i_). The network uses these logical addresses for routing.

### 5.2. Discovery Process

The discovery process is modeled using a breadth-first search (BFS) algorithm. It assigns a Virtual Routing Number (VRN) to each node. Let VRN:V→N be the function assigning these numbers. The BFS ensures that VRN(v_i_) < VRN(v_j_) for all v_i_ that are closer to the coordinator than v_j_. Discovery can be modeled as a graph traversal algorithm [13,42].

The discovery starts with the initialization step, where the coordinator node v0 is selected as the starting point. This node is pivotal in the network’s structure and is assigned as VRN(v_0_) = 0. Once the initialization is complete, the BFS algorithm takes over. This algorithm operates by exploring each node and its immediate neighbors in a systematic and layered approach, starting from the coordinator and moving outward.

For each node vi that the algorithm visits, it looks at its unvisited neighboring nodes, denoted as v_j_. These neighbors are the nodes directly connected to vi that have not been explored yet in the process. As each unvisited neighbor v_j_ is discovered, it is assigned a VRN that is one number higher than the VRN of the node from which it was discovered, vi. This is expressed as VRN(v_j_) = VRN(v*i*) + 1. This sequential assignment is a crucial aspect of the discovery process, as it indicates the node’s distance in terms of hops from the coordinator [15,19,39,43].

During the discovery process, the IQRF modules operate at a lower transmit power compared to normal network operations. For instance, if the standard communication uses level 7, the discovery might use a lower level like 6 or 5. This approach is designed to ensure that the discovered links are robust, as it prioritizes connections with closer and therefore stronger signal nodes. A lower transmit power level means that only nodes within the direct range of the coordinator or another node are discovered in each step. This method helps in building a network with stronger and more reliable links.

Throughout the process of discovery, specific zones are established. The definition of a zone Z_k_ is a set of vertices where each vertex v_i_ ∈ Z_k_ satisfies d(v_0_,v_i_) = k, where k is the number of hops from the coordinator [13,15,19].

### 5.3. Flooding Routing Mechanism

The flooding routing mechanism can be visualized as a wave of data transmission originating from the source node (coordinator) and progressively reaching all other nodes in the network. Each directed edge (v_i_,v_j_) in the graph is used for transmitting data but crucially, only during the time slots allocated to node v_i_ [13,19].

Let T:V→N be a function assigning time slots to each node. A message at node vi can be routed to v_j_ if T(v_i_) < T(v_j_). Each node vi is assigned specific time slots for transmission, based on its VRN. This is crucial for organizing transmissions in the network and preventing data collisions. When a node vi receives a message during its allocated time slot, it broadcasts this message to all its neighbors in the subsequent time slots allocated to it. This means that for each edge (v_i_,v*j*), the node v_i_ transmits the message to v_j_ only within its designated time slot. The message propagation in the network follows a sequential pattern. Initially, the message is broadcast from the coordinator node v_0_, reaching nodes directly connected to it (one hop away). In the following time slots, the message reaches nodes two hops away and the process continues, ensuring that the message eventually reaches all nodes in the network. The IQRF network basically utilizes the request–response pattern. This means that all the request messages are followed by a response message. The response messages at node v_j_ can be routed to v_i_ if T(v_j_) < T(v_i_). In the case of a response message, the direction of the message flow is changed as well as the timeslots [13,15,19].

The request message routing is represented as a directed acyclic graph, which is a subset of G where (v_i_,v_j_)∈E only if VRN(v_i_) < VRN(v_j_). The response is a subset of G where (v_i_,v*j*) ∈ E only if VRN(v_i_) > VRN(v_j_). In the case of request messages, the edges are directed from VRN(v_i_) → VRN(v_j_) where VRN(v_i_) < VRN(_vj_). The indegree of a vertex v in a directed graph is the number of edges coming into v.

In relation to the direction of message routing, the indegree and outdegree of nodes in the network can change. To make understanding and analyzing these degrees simpler, it is recommended to use the DAG that represents the routing of request messages as the basis for defining the in-degree and outdegree. Because of the way messages are routed, the response messages actually follow a path that is the transpose (or reverse) of the request message DAG. This means that the direction of edges in the DAG for response messages is opposite to that of the request messages, reflecting the reverse flow of information.

The indegree of a node v_i_ in the DAG, marked as deg^−^(v_i_), is the count of edges coming into v_i_ from other nodes. This number shows how many nodes can send information directly to v_i_. In a DAG organized by VRNs, a node vi with a specific VRN usually receives edges from nodes with lower VRNs. This means they are closer to the main control point in terms of network hops. Therefore, the indegree of vi depends on the number of nodes with lower VRNs nearby.

The outdegree of a node vi, marked as deg^+^(v_i_), is how many edges start at v_i_ and connect to other nodes. It shows the number of nodes vi that can send information directly. In the DAG, the outdegree of vi depends on the number of nodes with higher VRNs that it can reach. This indicates the node’s role in passing information further into the network.

The efficient availability of the adjacency matrix through FRC data collection makes it particularly useful for describing the indegrees and outdegrees of vertices in a graph. The network represented by an adjacency matrix A and the indegree and outdegree of a vertex, say vi, are determined by summing specific values in the matrix. The in-degree of vi, which indicates the number of edges entering this vertex, is calculated by adding up the entries in the ith column of the matrix. Similarly, the outdegree of v_i_, which counts the number of edges leaving the vertex, is found by summing the entries in the ith row of the adjacency matrix. Through these sums, the adjacency matrix provides a clear and structured way to analyze the connectivity of each vertex in the DAG [13,37].

### 5.4. Redundant Paths in the Network

A simple directed path within a graph G from vertex v_0_ to another vertex vi is defined as a sequence of vertices commencing at v*_0_* and concluding at vi. This sequence is characterized by two key features: firstly, each pair of consecutive vertices is linked by directed edges from the edge set E, and secondly, no vertex is revisited within the same path. A path is considered redundant if more than one distinct simple directed path exists leading from vertex v_0_ to vertex v_i,_ R(v_i_), which is the set of redundant simple directed paths from v_0_ to v_i_. Let P(v_i_) denote the set of all such simple directed paths from v_0_ to v_i_. If the cardinality of this set, ∣P(vi)∣, exceeds one, it indicates the presence of redundant paths for vertex vi originating from the coordinator vertex v_0_. A simple directed path in G from vertex 0 to another vertex v is a sequence of vertices starting at 0 and ending at v such that consecutive vertices are connected by directed edges in E and no vertex is repeated [25,41,42].

## 6. Network Communication Stability

Redundant connections can enhance the reliability of wireless sensor networks. Mathematically, this means that the degree of any node in a graph should be greater than one. However, in IQRF networks, packet repetition between two nodes is not feasible. Thus, redundant paths, through which messages can be received, are the only solutions to prevent data transmission failures. The degree of network redundancy in wireless networks can be characterized by the graph’s K-connectivity. The graph’s connectivity, κ(G), or its connectivity corresponds to the minimum number of nodes that, when removed, would fragment the network. This essentially quantifies how many nodes must become non-functional for the network to disintegrate. K-connectivity could be a viable metric for determining stability if the graph was considered undirected. However, during time-divided medium access-based flooding route selection, both request and response messages in the network have a defined direction, thus, the network can be modeled using a directed graph. The flooding route selection materializes through message repetition in the order of VRN numbers. Accordingly, the forwarding order of messages can only be in ascending order; hence, the graph is always a directed acyclic graph (DAG) [33,35,36,37,41].

**Figure 5:**
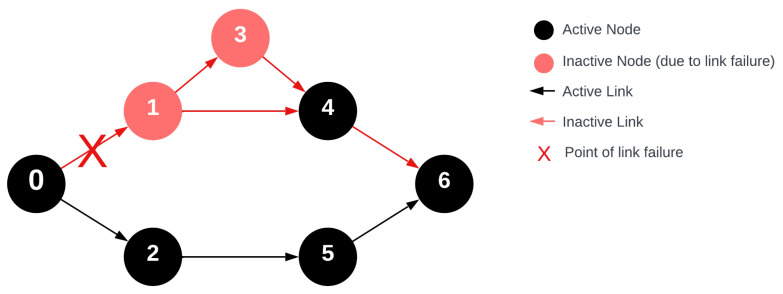
Wireless network with single network error during the routing request message.

Yet, there is not a metric that expresses the stability of the deployed network throughout its operation, considering the intricacies due to network redundancy. For the application of FRC data collection procedure, this paper proposes introducing a metric to gauge communication stability. This metric will evaluate the network’s capacity to tolerate connection failures and maintain stable communication. Figure 5 illustrates a network that showcases a fault between the coordinator and the u_1_ node. It is evident from the diagram that connectivity is not a suitable descriptor for stability, as the u_3_ node will not receive the message. While only one node is removed from the graph, the graph collapses. The u_4_ node receives the message because it can accept from the u_6_ node but it cannot repeat it as its dedicated time slot has already passed.

In our research, our primary objective was to establish a stability metric. This metric is designed to quantitatively evaluate the network’s capability to endure data transmission errors. Furthermore, it aims to indicate the maximum number of such errors the network can tolerate before its performance is adversely affected. Essentially, this stability metric serves as an analytical tool, allowing us to assess the network’s resilience and its threshold for maintaining optimal operations amidst transmission anomalies. This study discusses the main problems related to errors in communication links. We intentionally leave out probability-based methods for analyzing link failures from our research. Our main interest is in exploring how these network errors affect the stability of the whole network during data collection from the network. To explain these points, we show several example graphs and use them to gain a broader understanding of network stability.

## 7. Node Stability

**Figure 6:**
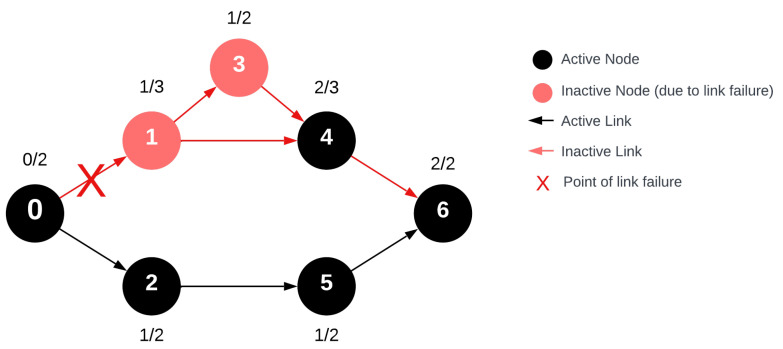
Degree/Indegree of vertices displayed above the network nodes.

Given that the network can be represented by a weakly connected directed graph, we assumed that the stability specific to nodes can define the overall stability of the entire network. Message transmission is based on the ascending order of VRN numbers. Therefore, a node can receive and forward a message from as many other nodes as there are with a VRN number lower than its own. In the graph model of the network, this corresponds to the node’s indegree, which we denote as de^−^(u). A node’s degree, on the other hand, determines from how many places it can receive the message, irrespective of its capability to forward the message. In Figure 6, each node’s indegree and degree are labeled above in the format deg^−^(u)/deg(u). By definition, since deg(u) = deg^−^(u) + deg^+^(u), if a node’s degree and indegree are determinable, its outdegree is derivable from this information [35,36].

From the figure, it can be inferred that if deg^−^(u) < 2 for any node, it implies that the node lacks redundant connections for message reception. Given the properties of time-divided message transmission, it is established that during the forwarding of the request broadcast message in the network, every node’s indegree cannot exceed its VRN number:

(1) $${deg}^{-}(u_{i})\leq VRN$$

The depicted data transmission error between u_0_ and u_1_ in the figure means that during message transmission, u_1_ and u_3_ will not receive the message since their indegrees are one and there is no alternative connection from which they can obtain the message.

(2) $$S_{i}={deg}^{-}(u_{i})$$

For any given node, we can infer that its stability, represented by the Si stability factor, corresponds directly to the node’s indegree. This indegree essentially captures the redundancy level of the node. To clarify, it denotes the number of devices from which a node can receive a message and still have the capability to forward it through the rest of the network. Due to the properties of the directed graph, a node can only receive messages from nodes with lower VRN values. Leveraging this understanding and framework for node stability provides valuable insights. These can be pivotal when devising protocols or shaping network structures that prioritize reliability, particularly in contexts where uninterrupted network communication is of paramount importance.

## 8. Network Stability

To evaluate the overall stability of the network, it is important to examine how each individual node impacts the entire network’s performance. The concept of network stability encompasses the following:

1. How robust the network is, demonstrating its ability to withstand and recover from disruptions. This can be expressed by a metric indicating the message delivery reliability of the network;
2. The total number of link failures the network can handle before it fails to deliver packets correctly.

**Figure 7:**
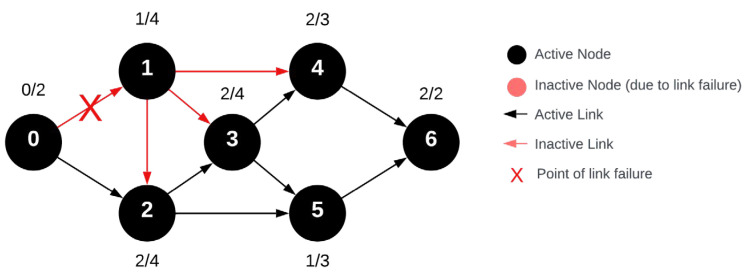
Modified wireless sensor network with single error during the routing request message.

A stable node implies that a coordinator has an indegree of zero, the largest indegree for u_1_ cannot exceed one, for u_2_ it cannot exceed two, and so forth. Since the indegree of nodes at the beginning of the network cannot surpass the VRN, their redundancy cannot be increased beyond VRN. The stability of an individual node was defined by the number of connections through which it can receive a message and still be capable of forwarding it. In Figure 7, the previously introduced network was altered by relocating the u_3_ vertex. In this modified network, all node indegrees changed to a minimum of two, except for u_1_. The previous network demonstrated that, in case of a data transmission error between the coordinator and u_1_ node, two nodes would drop out of the network. However, in the altered network, despite the same error, the message can reach every component of the network.

Successful message transmission occurred post-modification, even though the error occurred at the same location in the network. This is attributed to the message’s ability to reach all nodes via a different route. It is worth noting that while u_1_ does not fulfill the multiple redundancy criteria mentioned for node stability, it can still receive messages in this scenario. For example, in this case, after u_2_, u_3_ can receive the message. Therefore, even in the presence of an error, the rest of the network components can forward the message, fulfilling overall network stability and ensuring network redundancy. If we consider a singular error at any point in the network leads to a similar outcome, indicating that if any edge is terminated, the message can still reach every node.

From this analysis, we deduce that the overall network stability can be determined by aligning it with the minimum indegree of network nodes. However, the stability measure must correspond with nodes having an indegree not greater than the VRN. Otherwise, the stability would be defined by their minimum. In the example provided, the value S = 2 since min deg^−^(u) = 2 and for nodes with smaller VRN values, like u_1_ and u_2_, deg^−^(u) = VRN. If there was no edge between u_1_ and u_2_, then deg^−^(u_2_) = 1 and the network stability, Sn, would be 1. The coordinator acts as the source vertex and hence should not possess an indegree; it is irrelevant in terms of data transmission.

Let D^−^ = {deg^−^(u_0_), deg^−^(u_1_) … deg^−^(u_N_)} represent the indegrees of the graph’s vertices in the order of their VRN numbers. The stability factor that can be written for the network is

(3) $$S_{n}=\left\{\begin{array}{c} \min\;\{d_{i}\;|\;d_{i}\in D^{-},\;d_{i}<i,\;0\;\leq i<N\}\;\mathrm{if}\;\mathrm{such}\;\mathrm{di}\;\mathrm{exists}\\ \max\;\{d_{i}\;|\;d_{i}\in D^{-},\;0\;\leq i<N\}\;\mathrm{otherwise} \end{array}\right.$$

This conveys that S_n_ is the minimum of the set d_i_, where d_i_ is an element in D^−^, and less than its index i, with i being a natural number between 0 and N, representing the VRN number. If no such d_i_ exists, then S_n_ is the maximum of the set d_i_. This implies that the stability factor is determined by the smallest indegree among vertices whose indegree is smaller than their respective VRN numbers. It implies that in scenarios where vertices do exhibit indegrees lower than their VRNs, the network’s stability hinges upon the indegree of the first vertex that satisfies this condition, thereby providing a quantifiable measure of the network’s stability. This nuanced understanding of network stability, involving the relationship between indegrees and VRN, provides a foundation for evaluating and potentially enhancing the robustness and reliability of network communication paths.

The definition of stability factor S_n_ is determined by the weakest link in terms of the indegree relative to the VRN. In other terms, the stability of an IQRF network largely depends on its stability factor, S_n_, which is related to the concept of redundant paths. A redundant path in a network is an alternative route from one point, v_0_, to another, v_i_, that does not repeat any vertices along the route. The greater the number of such alternative paths, the more options the network has for sending data, enhancing its reliability.

The core idea from the proof is that the number of incoming connections (indegree) a node has is always less than or equal to the number of different ways data can reach it. In a network graph G, a redundant path is defined as a distinct simple directed path from a starting vertex v_0_ to another vertex v_i_. This path is characterized by two key features: first, each pair of linked vertices in this path is connected by directed edges, ensuring a clear direction from v_0_ to v_i_, and second, no vertex is revisited within the same path. A path is considered redundant if there is more than one distinct path from v_0_ to v_i_, forming a set R(v_i_) of such paths [39,41].

Each edge contributing to deg^−^(v_i_) represents a connection from some preceding vertex in the DAG to v_i_. These edges are the endpoints of paths originating from v_0_ and passing through intermediate vertices before reaching v_i_. If deg^−^(v_i_) > 1, it indicates that multiple edges enter v_i_, with each potentially being the end of a distinct path from v_0_. Since these paths originate from v_0_ and converge at v_i_, the presence of multiple such paths suggests the potential for redundancy. The maximum number of redundant paths ∣R(v_i_)∣ that v_i_ can have is equal to deg^−^(v_i_). In IQRF networks, where deg^−^(v_i_) ≥ i, the maximum indegree a node can have is i, where i is the VRN number of the given node [15,19].

In network topology, it is a well-established principle that the indegree of any given vertex does not surpass the number of redundant paths available to that vertex. This principle posits that the total number of incoming connections (indegree) at any network node is always equal to or less than the aggregate of distinct pathways through which data can be routed to this node [39,41,43].

This proof demonstrates that in all IQRF networks, where each node functions as a router, the stability factor S_n_ can indicate a minimum level of available redundancy in network connections to any node from the coordinator.

**Figure 8:**
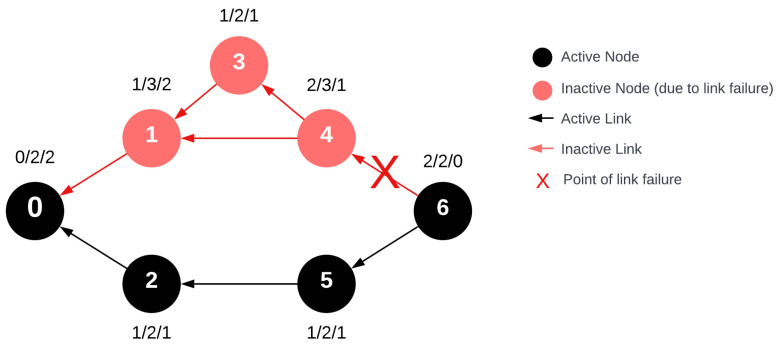
Wireless network with single link failure at node 4 during the routing response message affecting multiple nodes.

When a response message is sent, its flow is opposite to the initial request. The node degrees then transform such that the former indegree of a vertex will match its outdegree. In Figure 8, the graph formed during the response message transmission in the pre-modification network is displayed. For this graph, degree values corresponding to the response message’s progress are labeled above the nodes in the format deg^+^(u)/deg(u)/deg^−^(u).

**Figure 9:**
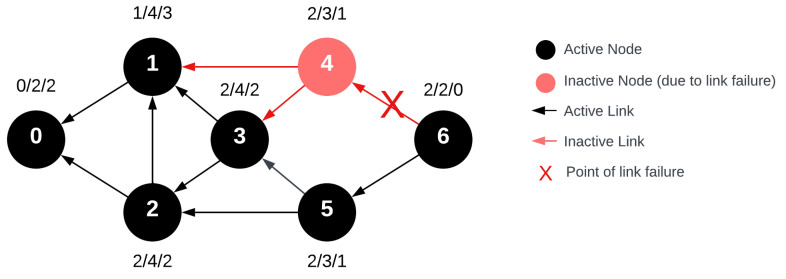
Modified network with single link failure at node 4 during the routing response message affecting a single node only.

Depending on the implementation, during the response message transmission, a node might be able to send its message even without receiving the prior response. This capability arises because, thanks to TDMA medium access, every node knows when its slot will come based on the packet size. Messages are concatenated during response forwarding; thus, even if one device fails, other devices might still transmit their response, regardless of whether they previously received the response message. Another implementation requires that a node must receive the response message before it concatenates and forwards them. From the standpoint of network stability, the latter is less advantageous and will be our focus. Figure 9 shows that during response message transmission, a single error can cause a significant portion of the network to drop out of message forwarding, mainly due to the absence of redundant paths. Analyzing the modified network’s behavior reveals that, despite the same error, only the affected node drops out from the message forwarding process and the rest of the network remains intact. The stability criterion written for the transmission process is also met during the response message transmission. For requests, the same criterion applies to message reception; for responses, it applies to transmission. This means that even if the same edge becomes terminated, it will not affect other parts of the network during the response message’s progression. The message can traverse the entire network despite the error’s presence.

In the event of a singular error during the request message transmission, one node might hear back from another, yet will not repeat if it has a smaller VRN number. This means it will not participate in route selection but will continue data collection if it receives the request from another node. However, during the response message transmission, using MDC might cause certain parts of the network to not relay data to the coordinator due to a single error. Nevertheless, thanks to FRC’s neighboring data transmission, data can still be delivered to the coordinator.

## 9. Stability of Data Collection

The network stability factor essentially outlines the resilience of network communication, indicating how many simultaneous network failures the system can withstand and still remain operational. Notably, in the context of the integrated data collection approach, the primary objective is ensuring that data from every network device reach the network coordinator, rather than guaranteeing data connection amongst all network elements. Data collection from the network can be compromised by any failure since any failure will likely result in nodes whose data do not reach the network coordinator. Taking this into account, a stability factor for data collection can be established. In the MDC process, this means that the stability factor must be one unit higher than the network’s fault tolerance since dual redundancy will only assure continued network operation after a single failure but can result in data loss. For the MDC procedure, the data collection stability factor can be written as

(4) $$S_{dMDC}=S_{n}-1$$

**Figure 10:**
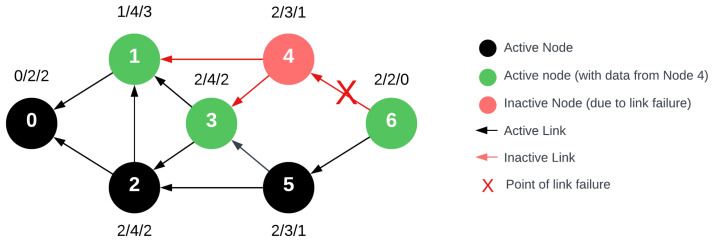
Modified network with single link failure at node 4 during the routing response message with FRC beacon data sharing. Notably, nodes 1, 3, and 6 (highlighted in green) successfully recorded data from node 4 during the beacon phase.

In the intermediate neighbor data sharing phase utilized by FRC, nodes transmit their data to every neighboring node. This implies that the data are received by deg(un) neighboring nodes. If the network satisfies S_n_ > 1, then at this phase, it holds true for every vertex that deg^−^(u_n_) > 1, meaning every node can share its data with at least two devices. From the standpoint of data collection stability, this denotes that in the event of a network failure, another node equipped with the missing data exists. The subsequent diagram, Figure 10, illustrates that if we assume the above-mentioned failure and S_n_ = 2 holds true, then during the FRC process, the points marked in green possess the data from the failed node, thereby preventing data loss.

Contrary to MDC, if the network meets the network stability criterion, then the desired level of data collection is achievable at the current stability. Accordingly, the data collection stability factor for the FRC process can be written as

(5) $$S_{dFRC}=S_{n}$$

The distinction from S_dMCD_ emanates predominantly from the beacon phase. This integration of an additional message transmission phase fortifies network stability by providing a more robust communication protocol.

By determining the stability factor, network designers and operators can evaluate the network’s reliable and continuous communication capability. A higher stability factor indicates a higher number of redundant connections within the network, thereby enhancing the network’s ability to withstand and recover from connectivity failures. Communication link stability in sensor networks is paramount for reliable data transmission and network connectivity. Network redundancy metrics, such as the number of redundant connections and redundant links, related to the data forwarding direction, are crucial for evaluating network stability. Research findings contribute to the design and operation of reliable wireless sensor networks, especially in chained data collection procedures. By determining the stability factor and evaluating the network’s redundancy, insights are gained into the design and development of wireless sensor networks, ensuring reliable and efficient data transmission for various applications.

## 10. Simulation of Packet Delivery Errors According to the Stability Factor

To verify the stability factor, we developed a simulation environment based on the model of the analyzed IQRF networks. These simulations were designed to demonstrate the impact of network link failures on the message delivery ratio, which is defined as the proportion of nodes that receive a broadcast message from the network coordinator. Given that IQRF network communication relies on flooding routing, every routing node repeats the broadcast message, which allows for measuring the message reach across nodes. In our simulations, each node functions as a routing node. When a link failure occurs and no redundant link is available to relay the message and the node is unable to receive a message, it is marked as a delivery error. We ran simulations for broadcast message delivery 10,000 times for each network. Link failures were simulated by randomly disabling a link during each timeslot of the routing process, aiming to reflect more realistic link failure scenarios. Over the course of the simulations, we gradually increased the number of link failures from 1 to 6 per timeslot. The stability factor is defined by how many link failures the nodes can withstand without incurring delivery errors.

**Figure 11:**
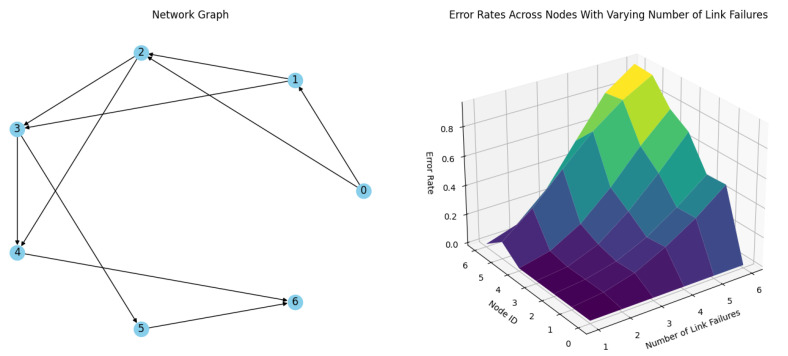
Simulation results of a network of 6 nodes and a coordinator with Sn = 1 stability factor with up to 6 link failures per timeslot.

**Simulation 1.** In the first simulation, the test network has a stability factor S_n_ = 1. Figure 11 illustrates the network and the packet delivery error rates for each node during 1 to 6 simultaneous link failures at each timeslot. As depicted in Table 1, the N5 node, which has fewer predecessors and a correspondingly lower indegree in the graph representation, contributes to the network’s stability factor being less than 2. This is also evident in the error rate results. N5 is the only node with a nonzero delivery error rate for the simulation with one failure. The limited redundancy in network links means that an increase in link failures leads to significantly higher delivery error rates. In scenarios where S_n_ = 1, the redundancy drops across the network, resulting in high delivery error rates at the nodes. Notably, Node 5 does not meet the redundancy criteria for S_n_ = 2, leading to a marked increase in delivery error rates from this node due to the lack of redundant links. Up to this point, the simulation results mirror those seen in the network depicted in Figure 11. The test simulation further demonstrates that the stability factor criteria can also highlight the weak points of the network.

**Figure 12:**
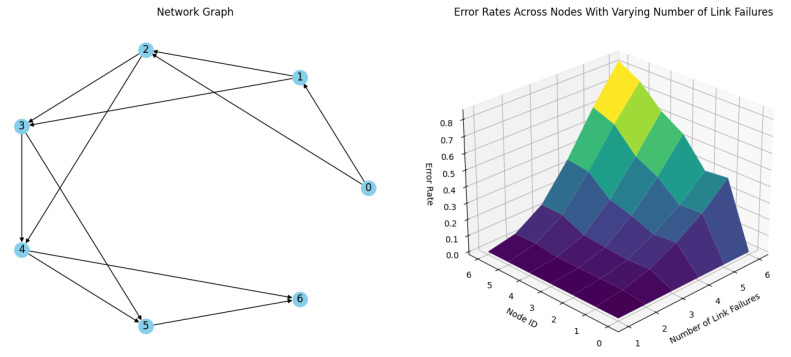
Simulation results of a network of 6 nodes and a coordinator with Sn = 2 stability factor with up to 6 link failures per timeslot.

**Simulation 2.** The next simulation, depicted on Figure 12, configures all nodes, including Node 5, with redundant links and achieves a stability factor of S_n_ = 2. The results demonstrate that with a single link failure, there are no delivery errors, confirming that the redundant links effectively ensure robust communication throughout the network with one random link failure per timeslot during routing. However, when the network experiences two simultaneous random link failures per timeslot, the delivery error rate remains low yet discernible. This highlights that while redundancy significantly bolsters network resilience, it is not entirely infallible. As the number of link failures increases, the delivery error rate climbs sharply, illustrating the constraints of redundancy in protecting against multiple concurrent failures, as depicted in Table 2.

**Figure 13:**
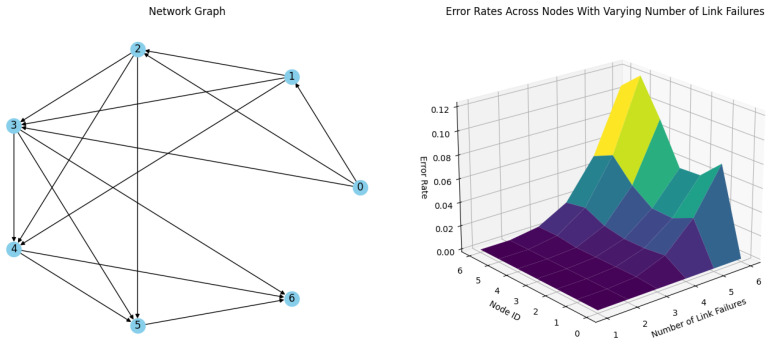
Simulation results of a network of 6 nodes and a coordinator with Sn = 3 stability factor with up to 6 link failures per timeslot.

**Simulation 3:** In this simulation, each node in the network was configured with up to three redundant links, achieving an S_n_ = 3 stability factor, as shown in Figure 13. This enhancement was anticipated to increase the packet delivery ratio and the results confirmed a substantial improvement in network performance, as illustrated in Table 3. With up to two simultaneous link failures, the network maintained perfect communication, successfully delivering all messages to every node without any errors. However, when the number of simultaneous link failures rose to three, some delivery errors occurred, though the error rates were significantly lower compared to the configuration with only two redundant links per node. This demonstrates that increasing redundancy to three links greatly enhances the network’s resilience to link failures, thereby ensuring higher reliability in message delivery, even under challenging conditions.

**Figure 14:**
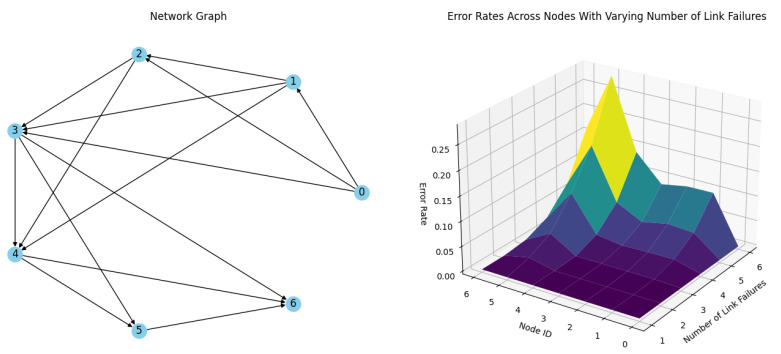
Simulation results of a network of 6 nodes and a coordinator with Sn = 2 stability factor modified from Sn = 3 by N5, which has only 2 predecessor node with up to 6 link failures per timeslot.

**Simulation 4.** Modifying the last simulation by decreasing the indegree of the N5 node resulted in a reduced stability factor from S_n_ = 3 to S_n_ = 2. This modification is evident in the simulation results shown in Figure 14 and Table 4. At two link failures per timeslot, the N5 node experienced delivery error rates, with higher error rates observed as the number of link failures increased. These results also illustrate how the stability factor effectively reflects the network’s communication stability.

**Figure 15:**
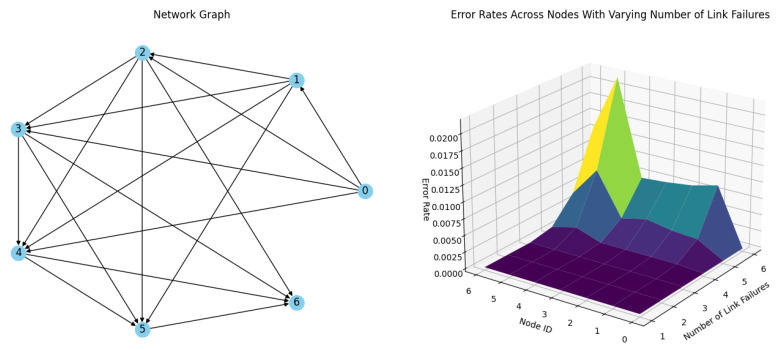
Simulation results of a network of 6 nodes and a coordinator with Sn = 4 with up to 6 link failures per timeslot.

**Simulation 5.** In the last simulation, the network’s stability factor was increased to S_n_ = 4. The results, as displayed in Figure 15 and Table 5, reveal no delivery errors for up to three random link failures per timeslot. Furthermore, a network with this high stability factor continues to exhibit low delivery errors, even with a greater number of link failures per timeslot. Despite the increased frequency of link failures, the error rates remained significantly lower than those observed in previous simulations. This highlights the substantial benefits of enhanced redundancy in boosting network resilience and ensuring consistent robust message delivery.

In summary, the simulation results have conclusively demonstrated that the stability factor serves as a robust indicator of network stability, especially in scenarios involving link failures during message routing. By quantifying the network’s ability to withstand and adapt to disruptions, the stability factor emerges as a critical metric for assessing the resilience of network communications. Additionally, the simulations highlight that the stability factor effectively reflects network stability through the impact of delivery error rates. Specifically, in networks with a stability factor of S_n_, there were no delivery errors for up to S_n_ − 1 link failures per timeslot.

## 11. Discussion

The detailed exploration of network stability, as discussed previously, requires careful analysis and interpretation. In assessing stability, the key is how well a network can keep communicating, particularly when facing multiple failures. The approaches of MDC and FRC offer interesting contrasts. For MDC, the stability factor must be one higher than the network’s link failure tolerance to ensure uninterrupted operation. This highlights a hidden risk: a single failure may not disrupt communication but could result in data loss. In contrast, FRC focuses on data sharing between neighboring nodes, promoting data redundancy and thus resilience. If each node shares its data with at least two others, the network gains a safety net against disruptions.

However, the concept of the stability factor goes beyond just handling faults or creating redundancies. It is a valuable tool for network designers and operators to measure a network’s ability to maintain consistent and reliable communication. A higher stability factor suggests more redundant connections, enhancing the network’s defense against connectivity issues. The importance of stable communication links in sensor networks is particularly critical, given their role in ensuring reliable data transmission and network connectivity. Factors like the number of redundant links and their alignment with data forwarding paths greatly enhance our understanding of how to evaluate network stability.

To demonstrate that the stability factor S_n_ is universally applicable across all IQRF networks, it is essential to look into the specifics of its definition and implications. The stability factor S_n_ is defined as the minimum indegree among vertices with an indegree less than their respective VRN. In essence, this implies that every network node involved in message routing should have at least S_n_ redundant routes from the coordinator v_0_ to a specific node v_i_.

The concept of indegree in a directed graph is straightforward: it refers to the count of incoming edges to a vertex v. A redundant path from vertex 0 to vertex v is a simple directed path, one of at least two distinct simple directed paths leading from 0 to v.

Let us consider any vertex v in a directed graph G. We denote the indegree of v as deg^-^(v) and the set of redundant simple directed paths from vertex 0 to v as R(v). We aim to establish that deg^-^(v) ≤ ∣R(v)∣ [25,41,42].

By analyzing each edge that leads into vertex v, we observe that

- If a path from 0 to v exclusively utilizes a specific incoming edge to v, this path is not redundant, as it lacks an alternative route involving that edge;
- Conversely, if an incoming edge to v is shared by multiple paths from 0 to v, each of these paths qualifies as redundant, given the presence of another path utilizing the same edge [25,41];
- Therefore, every incoming edge to v contributes to at least one path in R(v). While some edges may be part of the same path in R(v), none can contribute to a path outside R(v) without rendering that path redundant;
- Consequently, the total count of edges incoming to v (i.e., deg^−^(v)) cannot exceed the number of redundant paths in R(v), as each edge factors into the total of ∣R(v)∣ [25,41].

This analysis establishes that deg^−^(v) ≤ ∣R(v)∣, confirming that the indegree of any vertex v in the graph is always less than or equal to the number of redundant simple directed paths from vertex 0 to v. This conclusion is predicated on the characterization of redundant paths as possessing at least two distinct routes that share a minimum of one common edge.

Regarding network stability, both link and node failures affect it. However, node failures are more critical since they disrupt the node and all connected links, potentially having a more significant impact than a single link failure. The existence of multiple distinct paths in the network ensures that traffic can still be rerouted if a path becomes unusable due to a link or node failure. In a directed graph, a node failure can be represented as the failure of all incoming and outgoing links for that node [25,42,44]. In IQRF networks, where nodes are ordered, a failure at node v*i* impacts subsequent nodes v_j_ (where j > i), leading to a lower indegree for these nodes. As established, the indegree of any vertex v in the graph is limited by the number of redundant directed paths from 0 to v. If a node failure affects any path in the set R(v) between vertices 0 and v, it impacts only one path, reducing ∣R(v)∣ [25,41]. However, if ∣R(v)∣ > 0 post-failure, the network remains operational. The network’s stability factor S_n_ indicates its capacity to endure up to S_n_ − 1 node failures, assuming at least S_n_ redundant routes from the coordinator v_0_ to a specific node v_i_.

The stability factor can be a useful metric in designing network installations by providing insights into achievable stability. This metric aids in planning; for instance, if the installation environment requires increased redundancy in communication links, the stability factor can guide the placement of network devices to achieve the desired stability.

The simulation results demonstrate that the stability factor effectively indicates network stability through the impact of delivery error rates. In networks with a stability factor of S_n_, there were no delivery errors for up to S_n_−1 link failures per timeslot. Generally, a stability factor (S_n_) greater than 1 is sufficient, but this depends on the specific application. Post-installation, the network’s adjacency matrix can be efficiently mapped, allowing for the reconstruction of the network topology. The stability factor is calculated based on the indegrees of the nodes. Nodes with an indegree smaller than their VRN are easily identifiable. Increasing the indegree of these nodes depends on the feasibility of relocation strategies. While adding more routing devices is an option, it can lead to longer data collection times and increased battery usage.

Taken together, these findings enhance our comprehension of IQRF wireless sensor network design and operation, particularly in the context of concatenated data collection procedures. The marriage of stability factor determination and network redundancy assessment offers a robust foundation for the design and development of these networks, ensuring reliable and efficient data transmission across diverse applications. For future research, diving deeper into the relationships and links between nodes, especially within inhomogeneous networks, may unveil additional dimensions to network stability and optimization.

## 12. Conclusions

In the field of network communication, the understanding and quantification of IQRF network stability is crucial. This study thoroughly examined how the stability factor influences the resilience of network communications, particularly against multiple simultaneous network failures. The key findings highlight the importance of ensuring data from each network device reaches the network coordinator, even more so than maintaining connectivity among all network elements. Two pivotal data collection approaches were discussed in detail: the merged data collection (MDC) and the IQRF’s Fast Response Command (FRC). The basic MDC process, with its stability factor being one unit higher than the network’s fault tolerance, underscores the significance of redundancy. This ensures continued operation post failures but warns of potential data losses, highlighting a clear area for future improvements. In contrast, the FRC method offers a more holistic approach, where nodes are equipped with redundant data, thereby substantially reducing the risks associated with data loss during network failures.

A key insight from this study is the undeniable correlation between the stability factor and network redundancy. The higher the stability factor, the greater the network’s resilience to connectivity disruptions. Simulation results confirmed that the stability factor effectively represents network stability against link failures during message routing. Such an insight provides invaluable guidance for network designers and operators, urging them to invest in mechanisms that augment the stability factor. Furthermore, the research underscores the crucial role of communication link stability in the IQRF sensor networks. A stable communication link is the bedrock for reliable data transmission and seamless network connectivity. By integrating network redundancy metrics, such as redundant connections and links, with the data forwarding direction, one can achieve a comprehensive evaluation of network stability.

## Figures and Tables

**Table 1:** Packet delivery error rates of simulation results of a network of 6 nodes and a coordinator with Sn = 1 stability factor with up to 6 link failures per timeslot.

Node #	1 Failures	2 Failures	3 Failures	4 Failures	5 Failures	6 Failures
Node 1	0.00%	**2.39%**	**8.13%**	**18.53%**	**31.37%**	**47.35%**
Node 2	0.00%	**2.39%**	**7.41%**	**16.97%**	**30.59%**	**46.53%**
Node 3	0.00%	**2.39%**	**10.78%**	**26.62%**	**46.41%**	**68.35%**
Node 4	0.00%	**4.83%**	**16.69%**	**35.30%**	**57.57%**	**76.79%**
Node 5	**9.88%**	**26.53%**	**47.05%**	**66.56%**	**81.95%**	**93.28%**
Node 6	0.00%	**6.75%**	**25.16%**	**52.73%**	**79.92%**	**94.31%**

**Table 2:** Packet delivery error rates of simulation results of a network of 6 nodes and a coordinator with Sn = 2 stability factor with up to 6 link failures per timeslot.

Node #	1 Failures	2 Failures	3 Failures	4 Failures	5 Failures	6 Failures
Node 1	0.00%	**1.25%**	**4.18%**	**7.97%**	**14.36%**	**24.10%**
Node 2	0.00%	**1.25%**	**4.18%**	**7.89%**	**13.60%**	**23.12%**
Node 3	0.00%	**1.25%**	**4.18%**	**8.62%**	**17.00%**	**30.70%**
Node 4	0.00%	**2.59%**	**9.36%**	**20.09%**	**33.21%**	**49.72%**
Node 5	0.00%	**1.25%**	**5.36%**	**11.48%**	**22.01%**	**35.92%**
Node 6	0.00%	**1.25%**	**4.45%**	**10.34%**	**21.59%**	**38.66%**

**Table 3:** Packet delivery error rates of simulation results of a network of 6 nodes and a coordinator with Sn = 3 stability factor with up to 6 link failures per timeslot.

Node #	1 Failures	2 Failures	3 Failures	4 Failures	5 Failures	6 Failures
Node 1	0.00%	0.00%	**0.16%**	**1.12%**	**3.48%**	**7.30%**
Node 2	0.00%	0.00%	**0.16%**	**0.92%**	**2.69%**	**5.60%**
Node 3	0.00%	0.00%	**0.16%**	**0.92%**	**2.76%**	**5.50%**
Node 4	0.00%	0.00%	**0.16%**	**1.25%**	**4.01%**	**9.02%**
Node 5	0.00%	0.00%	**0.39%**	**2.06%**	**5.90%**	**12.11%**
Node 6	0.00%	0.00%	**0.38%**	**1.75%**	**5.12%**	**10.61%**

**Table 4:** Packet delivery error rates of simulation results of a network of 6 nodes and a coordinator with Sn = 2 stability factor modified from Sn = 3 with up to 6 link failures per timeslot.

Node #	1 Failures	2 Failures	3 Failures	4 Failures	5 Failures	6 Failures
Node 1	0.00%	0.00%	**0.20%**	**1.37%**	**4.06%**	**9.59%**
Node 2	0.00%	0.00%	**0.20%**	**1.31%**	**4.40%**	**9.50%**
Node 3	0.00%	0.00%	**0.20%**	**1.13%**	**3.30%**	**8.55%**
Node 4	0.00%	0.00%	**0.20%**	**1.86%**	**5.93%**	**13.86%**
Node 5	0.00%	**1.17%**	**3.33%**	**9.01%**	**16.26%**	**28.25%**
Node 6	0.00%	0.00%	**0.53%**	**2.43%**	**7.04%**	**16.44%**

**Table 5:** Packet delivery error rates of simulation results of a network of 6 nodes and a coordinator with Sn = 4 stability factor with up to 6 link failures per timeslot.

Node #	1 Failures	2 Failures	3 Failures	4 Failures	5 Failures	6 Failures
Node 1	0.00%	0.00%	0.00%	**0.03%**	**0.22%**	**0.87%**
Node 2	0.00%	0.00%	0.00%	**0.03%**	**0.25%**	**0.77%**
Node 3	0.00%	0.00%	0.00%	**0.03%**	**0.32%**	**0.72%**
Node 4	0.00%	0.00%	0.00%	**0.03%**	**0.23%**	**0.69%**
Node 5	0.00%	0.00%	0.00%	**0.16%**	**0.88%**	**2.16%**
Node 6	0.00%	0.00%	0.00%	**0.06%**	**0.42%**	**1.29%**

## Data Availability

Data is contained within the article.
